# Nationwide Experience of Treatment with Protease Inhibitors in Chronic Hepatitis C Patients in Denmark: Identification of Viral Resistance Mutations

**DOI:** 10.1371/journal.pone.0113034

**Published:** 2014-12-01

**Authors:** Christina Sølund, Henrik Krarup, Santseharay Ramirez, Peter Thielsen, Birgit T. Røge, Suzanne Lunding, Toke S. Barfod, Lone G. Madsen, Britta Tarp, Peer B. Christensen, Jan Gerstoft, Alex L. Laursen, Jens Bukh, Nina Weis, The DANHEP group

**Affiliations:** 1 Department of Infectious Diseases, Copenhagen University Hospital, Hvidovre, Denmark; 2 Copenhagen Hepatitis C Program (CO-HEP), Department of Infectious Diseases and Clinical Research Centre, Copenhagen University Hospital, Hvidovre, Denmark; and Department of International Health, Immunology and Microbiology, Faculty of Health and Medical Sciences, University of Copenhagen, Copenhagen, Denmark; 3 Section of Molecular Diagnostics, Clinical Biochemistry and Department of Medical Gastroenterology, Aalborg University Hospital, Aalborg, Denmark; 4 Department of Gastroenterology, Copenhagen University Hospital, Herlev, Denmark; 5 Department of Medicine, Kolding Hospital, Kolding, Denmark; 6 Department of Infectious Diseases, Copenhagen University Hospital, Hillerød, Denmark; 7 Department of Infectious Diseases, Roskilde Hospital, Roskilde, Denmark; 8 Department of Gastroenterology, Køge Hospital, Køge, Denmark; 9 Diagnostic Center, Silkeborg Regional Hospital, Silkeborg, Denmark; 10 Department of Infectious Diseases, Odense University Hospital, Odense, Denmark; 11 Department of Infectious Diseases, Copenhagen University Hospital, Rigshospitalet, Copenhagen, Denmark; 12 Department of Infectious Diseases, Aarhus University Hospital, Skejby, Aarhus, Denmark; 13 Department of Clinical Medicine, Faculty of Health and Medical Sciences, University of Copenhagen, Copenhagen, Denmark; Inserm, U1052, UMR 5286, France

## Abstract

**Background and Aims:**

The first standard of care in treatment of chronic HCV genotype 1 infection involving directly acting antivirals was protease inhibitors telaprevir or boceprevir combined with pegylated-interferon and ribavirin (triple therapy). Phase III studies include highly selected patients. Thus, treatment response and development of viral resistance during triple therapy in a routine clinical setting needs to be determined. The aims of this study were to investigate treatment outcome and identify sequence variations after triple therapy in patients with chronic HCV genotype 1 infection in a routine clinical setting.

**Methods:**

80 patients, who initiated and completed triple therapy in Denmark between May 2011 and November 2012, were included. Demographic data and treatment response were obtained from the Danish Database for Hepatitis B and C. Direct sequencing and clonal analysis of the RT-PCR amplified NS3 protease were performed in patients without cure following triple therapy.

**Results:**

38 (47%) of the patients achieved cure, 15 (19%) discontinued treatment due to adverse events and remained infected, and 27 (34%) experienced relapse or treatment failure of whom 15 of 21 analyzed patients had well-described protease inhibitor resistance variants detected. Most frequently detected protease variants were V36M and/or R155K, and V36M, in patients with genotype 1a and 1b infection, respectively.

**Conclusions:**

The cure rate after triple therapy in a routine clinical setting was 47%, which is substantially lower than in clinical trials. Resistance variants towards protease inhibitors were seen in 71% of patients failing therapy indicating that resistance could have an important role in treatment response.

## Introduction

Persistent hepatitis C virus (HCV) infection is a major cause of chronic hepatitis, cirrhosis and hepatocellular carcinoma. Liver related mortality, once cirrhosis has developed, is 3% per year [1]. Until recently, standard-of-care (SOC) for chronic HCV genotype (GT) 1 infection was pegylated-interferon alfa (PEGINF) and ribavirin (RBV), curing only 40–50% of patients (Pt.) [2]. Protease inhibitors (PI's) telaprevir (TEL) and boceprevir (BOC), targeting the HCV non-structural 3 (NS3) protease, have, when added to PEGINF/RBV (triple therapy), improved cure rates up to 79% in clinical trials of highly selected, treatment-naïve and relapse HCV GT1 infected patients [3]. However, due to possible selection bias it is not yet clear if this promising treatment response can be transferred into a routine clinical setting.

In chronic HCV infection, the circulating viral quasispecies can harbour PI resistant variants, identified in 0.2 to 2.8% of treatment naïve patients [4]. Under pressure of antiviral treatment, PI resistant variants are selected in patients with no or partial response to treatment. Several major amino acid (aa) positions within the NS3 HCV protease [5]–[7] associated with different resistance levels have been identified, and most confer broad cross-resistance between TEL and BOC [1], [8]. The selection, emergence and persistence of PI resistant viruses are of significant concerns, since these resistant variants could influence future treatment options with second generation PI's.

The objectives of this study were to investigate treatment response to triple therapy in HCV GT1 patients in a nationwide, routine clinical setting, and to describe the development of variations in the protease sequence after treatment failure.

## Materials and Methods

### Patients and treatment

Study participants recruited from The Danish Database for Hepatitis B and C (DANHEP) commenced triple therapy from May 2011 to November 2012. They were treated in accordance with Danish guidelines [9], [10].

Investigators prescribed treatment following the manufacturer's protocol of the relevant PI. Outcome was defined as sustained virological response (SVR; negative HCV-RNA 24 weeks after End of Treatment (EOT)), relapse (undetectable HCV-RNA at treatment completion, but detectable during follow-up), viral breakthrough (HCV-RNA levels initially decreases during treatment (undetectable levels can be seen), followed by a clinical relevant increase while on treatment) or non-response (persistent HCV-RNA positive) [5].

Demographic data, choice of PI, RBV dose reduction and triple therapy response (**Table 1**) were extracted from DANHEP. The HCV subtype was determined by RT-PCR amplification and direct sequencing [11]–[13].

**Table 1:** Baseline characteristics of the HCV patients completing triple therapy.

		*Total*	*SVR*	*Non-SVR*	*P-value*
		*N = 80* *	38 (47%)	42 (53%)	
Male sex		47	27 (57%)	20 (43%)	0,058
Age	≤45 years	20	11 (55%)	9 (45%)	
	>45 years	60	27 (45%)	33 (55%)	0,605
Ethnicity	White	64	31 (48%)	33 (52%)	0,955
	Other	16	7 (44%)	9 (56%)	
Mode of	IDU	34	14 (41%)	20 (59%)	0,602
transmission					
	Non-IDU	13	6 (46%)	7 (54%)	
	Unknown	33	18 (55%)	15 (45%)	
Mild fibrosis		17	9 (53%)	8 (47%)	
Moderate fibrosis		30	19 (63%)	11 (37%)	0,029
Cirrhosis		33	10 (30%)	23 (70%)	
SOC treatment	Naïve	44	25 (57%)	19 (43%)	
experience					
	Relapse	16	7 (44%)	9 (56%)	0,137
	Non-response	20	6 (30%)	14 (70%)	
IL-28B genotype	C/C	14	10 (71%)	4 (29%)	
	C/T	37	20 (54%)	17 (46%)	0,010
	T/T	16	3 (19%)	13 (81%)	
	Unknown+	13	5 (38%)	8 (62%)	
HIV/Hepatitis B		9	4 (44%)	5 (56%)	1
virus co-infection					
HCV-RNA level at	>600.000 IU/ml	54	25 (46%)	29 (54%)	0,943
baseline	<600.000 IU/ml	26	13 (50%)	13 (50%)	
ALT level at	≥2 × UNL++	22	10 (45%)	12 (55%)	1
baseline	<2 × UNL	58	28 (48%)	30 (52%)	
Subtype**	1a	53	26 (49%)	27 (51%)	0,997
	1b	26	12 (46%)	14 (54%)	
Protease inhibitor	Telaprevir	38	14 (37%)	24 (63%)	0,112
	Boceprevir	42	24 (57%)	18 (43%)	
Ribavirin dose	Yes	32	16 (50%)	16 (50%)	0,891
reduction	No	48	22 (46%)	26 (54%)	

*1 patient was lost to follow-up at treatment week 16.

**One patient was infected with HCV genotype 1l.

+ No material available in DANHEP for IL-28B genotyping.

++ Upper Normal Level (50 U/L).

SOC; standard of care, SVR; sustained viral response, IDU; intravenous drug use.

### Blood samples

Blood samples were taken at baseline, during treatment weeks 1, 2, 4, 5, 6, 8, 12, 24, 36 and 48 for BOC and 1, 2, 4, 8, 12, 24, 36, 48 for TEL, and at weeks 12, 24 and 48 post-treatment. HCV-RNA titres (**Table 2**) were measured at baseline, at treatment weeks 4, 8, 12, 16, 24, 28, 36, 40 and 48, and 12 and 24 weeks after treatment withdrawal. Analyses of the NS3 protease were performed on baseline and post-treatment samples from non-responders or patients with viral breakthrough/relapse. Patients 7, 14 and 21 had no bio-bank sample at treatment failure, thus blood samples taken later were analyzed.

**Table 2:** HCV-RNA titres through the course of treatment for 21 non-SVR patients analyzed for protease sequence variations.

Patient ID	GT	TEL/BOC	HCV-RNA titre baseline (IU/ml)	HCV-RNA titre Week 4 (IU/ml)	HCV-RNA titre Week 8 (IU/ml)	HCV-RNA titre Week 12 (IU/ml)	HCV-RNA titre Week 16 (IU/ml)	HCV-RNA titre Week 24 (IU/ml)	HCV-RNA titre Week 40 (IU/ml)	HCV-RNA titre Week 48 (IU/ml)
1	1a	TEL	5320000	<15	Negative	Negative	Negative	Negative	**24100**	NT
2	1a	TEL	82700	<15	NA	Negative	NA	**2390**	NT	NT
3	1a	TEL	5300000	Positive	35	Negative	Negative	Negative	**570000 (36)**	NT
4	1a	TEL	2600000	850	**4600**	NT	NT	NT	NT	NT
5	1a	TEL	23000000	500	Negative	40	400	**2700000**	NT	NT
6	1a	TEL	10000000	<15	100	**2300**	NT	4600	NT	NT
7	1a	TEL	2300000	1000	80000	NT	NT	NT	NT	**EOT (56) 270000**
8	1a	BOC	900000	420000	2000	**500000**	NT	NT	NT	NT
9	1a	BOC	2500000	470000	1400	Positive	Positive	Positive	**3400000**	NT
10	1a	BOC	12000000	1600	<15	6000	600	80	NT	**2300000**
11	1a	BOC	48000000	5200	900	**4800**	NT	NT	NT	NT
12	1a	BOC	2200000	9000	Negative	Negative	Negative	Negative	Negative	**14000000**
13	1a	BOC	280000	3000	Negative	Negative	Negative	Negative	**430000 (29)**	NT
14	1a	BOC	1400000	550000	6000	1000	NT	NT	NT	**EOT (48) 3800000**
15	1b	TEL	2200000	**670000**	NT	NT	NT	NT	NT	NT
16	1b	TEL	139000	<50	<50	<50	**249000**	NT	NT	NT
17	1b	TEL	6200000	Negative	Negative	Negative	**100000**	NT	NT	NT
18	1b	TEL	5790000	15	NT	Negative	NA	**174000**	NT	NT
19	1b	BOC	630000	42000	180	130	NT	**EOT (24) 330000**		
20	1b	BOC	20000000	1100000	80	Negative	Negative	Negative	Negative	**EOT (12) 1900000**
21	1b	BOC	6500000	Negative	Negative	Negative	Negative	Negative	Negative	**EOT (48) 440000**

Sample analyzed for resistance is marked in bold.

SVR; sustained viral response.

TEL; telaprevir, BOC; boceprevir

NT; Not taken.

NA; Not available.

GT; genotype. EOT; End of treatment.

(12) week 12, (24) week 24, (29) week 29, (36) week 36. (48) week 48, (56) week 56.

HCV-RNA levels were measured with a real-time TaqMan assay (Roche Molecular systems) with a lower limit of detection of 15 IU/ml [14] or an in-house real-time PCR method as described [15]. IL-28B genotyping was performed as described [16].

### Genotype, Phylogenetic and Sequence Analysis

**Figure 1:**
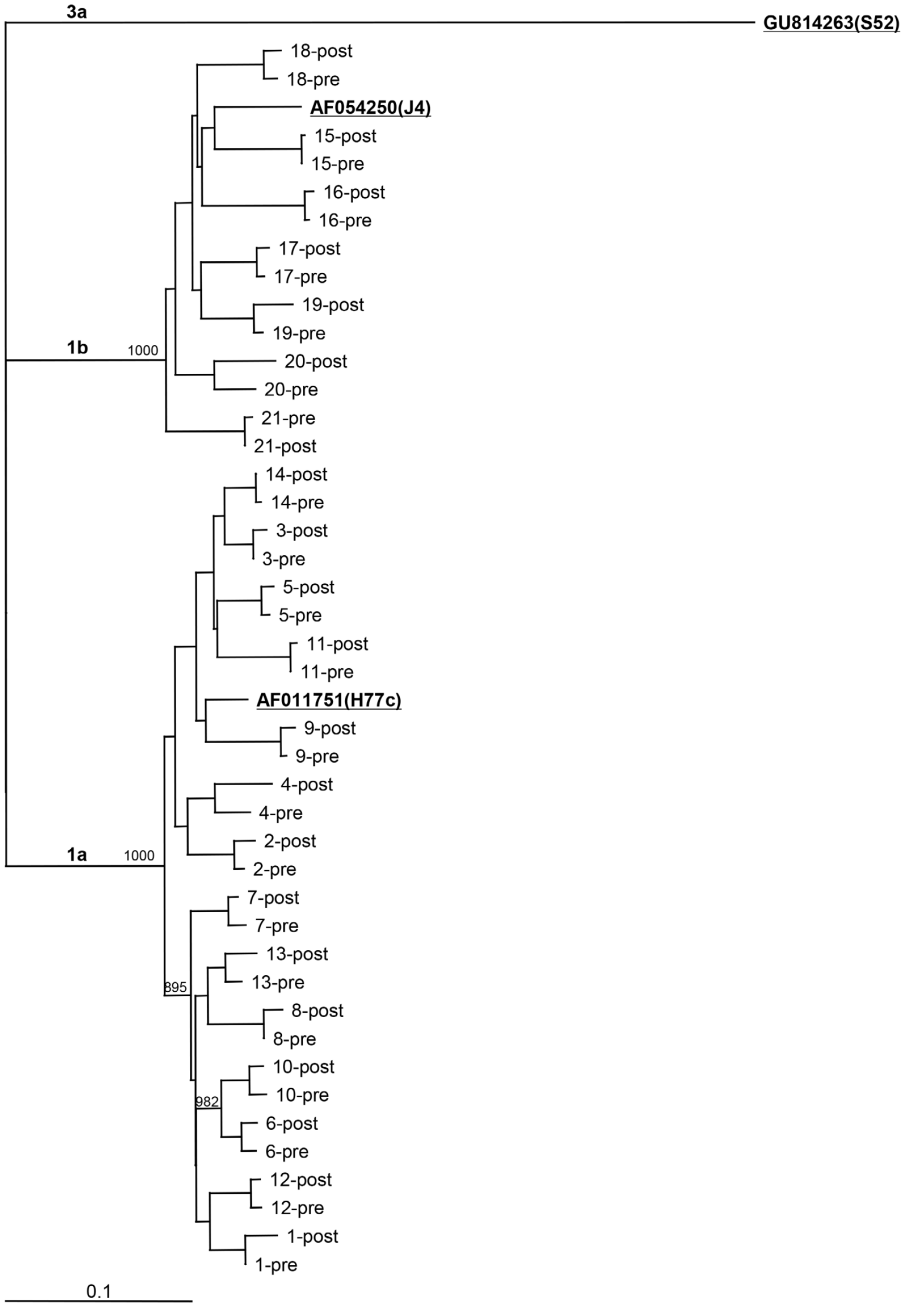
Phylogenetic tree of NS3-protease nucleotide sequences obtained by direct sequencing of pre- and post-treatment samples, for 21 selected genotype 1 patients with non-SVR analyzed for resistance variants. The tree was constructed by Neighbor-Joining with Kimura 2-parameter as the nucleotide substitution model. Support of nodes was based on bootstrap analysis (1000 replicates); only values over 850 are represented. The bottom horizontal bar represents 0.1 substitutions per nucleotide position. Reference strains for genotype 1a (H77C, AF011751), genotype 1b (J4, AF054250), and genotype 3a (S52, GU814263) are shown in bold and underlined. Genotype 3a, S52 was used as an out-group and root of the tree. Patients were grouped according to their genotype, 1a (n = 14), or 1b (n = 7). As expected, the pre- and post-samples taken from each individual patient clustered together, and bootstrap values of all final nodes were >850. No other significant clustering was observed.

Viral HCV-RNA was extracted from 200 µl of plasma or serum by using High Pure Viral RNA kit from Roche. For non-SVR patients to be analyzed for protease variants, the subtype was confirmed in the pre-treatment sample by analysis of C-E1 amplicons [11]. For NS3 protease analysis, direct sequencing of RT-nested PCR amplicons was done in 21 non-SVR patients; six patients, one with HCV GT 1l and five without study post-treatment samples were not included. RT was performed with Superscript III (Invitrogen) and cDNA was treated with RNAse H and T1. First round and nested PCR were performed with standard cycling conditions, using *Taq*Gold polymerase (Applied Biosystems) for low viral load samples and Advantage (Clontech) for high viral load samples (primers are listed in **Table 3**). DNA was column-purified (Wizard Genomic DNA Purification Kit, Promega) and sequenced (Macrogen Inc.), and a phylogenetic tree (**Fig. 1**) was generated for intra-individual confirmation. In a subgroup of 14 patients, NS3 amplicons at baseline and post-treatment were subjected to TA cloning (TOPA TA Cloning kit, Invitrogen). NS3 sequences, obtained pre- and post-treatment, were inspected for presence of putative resistant variants by Sequencher version 5.1 (Gene Codes Corporation) and Bioedit (http://www.mbio.ncsu.edu/BioEdit/bioedit.html) (**Table 4**) [1], [4], [7], [17], [18].

**Table 3:** Primer combinations used for amplification of the NS3 protease.

	Forward		Reverse		
Procedure	H77 position	Sequence 5′- 3′	H77 position	Sequence 5′- 3′	Amplicon size (nt*)
RT			4263	CTCCTGAGCACCCGCCGTC	
PCR	3150	GCCATCATCAAGTTAGGGGCG	4263	CTCCTGAGCACCCGCCGTC	1114
Nested-PCR	3160	AGTTAGGGGCGCTTACTGGCAC	4241	AAGGAACTTGCCGTAGGTGGAGTA	1082
RT			5659	TGTATCCCACTGATGAAATTCCAC	
PCR	3163	TAGGGGCGCTTACTGGCAC	5659	TGTATCCCACTGATGAAATTCCAC	2497
Nested-PCR	3173	TACTGGCACCTATGTGTATAACCA	5649	TGATGAAATTCCACATGTGCTTCG	2477
RT			6796	TGGAGTCCTACTCTGAATGATACC	
PCR	2728	TCCTCCTGCTCCTGCTGGCGTTGC	5137	GCTTGAGCCCTAGCGCACACGGTG	2410
Nested-PCR	3165	GGGGCGCTTACTGGCACCTATG	4329	CGATGCCCAAGATGGATGTGG	1165
	J4 position	Sequence 5′- 3′	J4 position	Sequence 5′- 3′	
RT			5650	TGATGAAATTCCACATGTGCTTCG	
PCR	3262	GTCGTCTTCTCCGACATGGA	5650	TGATGAAATTCCACATGTGCTTCG	2389
Nested-PCR	3275	ACATGGAGACCAAGGTCATCAC	5644	AATTCCACATGTGCTTCGCCCA	2370
RT			4306	CAGTTGAGTGGCACTCATCACA	
PCR	3275	ACATGGAGACCAAGGTCATCAC	4306	CAGTTGAGTGGCACTCATCACA	1032
Nested-PCR	3292	ATCACCTGGGGGGCAGATACCG	4289	TCACATATTATGATGTCATAGG	998
RT			5507	ATCCCCTGCTCGATGTAAGG	
PCR	3275	ACATGGAGACCAAGGTCATCAC	5507	ATCCCCTGCTCGATGTAAGG	2233
Nested-PCR	3292	ATCACCTGGGGGGCGGACACCG	5472	CTCTTCCATTTCATCGAACTCC	2181
RT			5510	TGCATTCCCTGTTCGATGTATGG	
PCR	3275	ACATGGAGACCAAGGTCATCAC	5510	TGCATTCCCTGTTCGATGTATGG	2236
Nested-PCR	3292	ATCACCTGGGGGGCGGACACCG	5500	TCGATGTATGGGAGGTGCG	2209
RT			6547	GTCCCGTGGTGTATGCGTTGATGG	
PCR	2045	CGTGTAACATCGGGGGGGTCGG	5526	AAATTGCTCGGCGAGCTGCATTCC	3482
Nested-PCR	2772	GGACCGGGAGATGGCTGCATCGTG	4629	GATAGGCGGTATGACGGACAC	1858

*Nucleotide.

The primer names refer to the full genome nucleotide locations on reference strains H77 (AF009606) and J4 (AF054249).

**Table 4:** Resistance protease variants identified in vitro in replicon-based studies and in vivo in clinical trials [1], [4], .

NS3 residue	In vivo	In vitro	TEL	BOC
V36	A,G,L,M	A,G,L,M	X	X
Q41	R	R	X	X
F43	S	S	X	X
T54	A,S	A	X	X
V55	A, I, F		X	X
Q80	K,L,N			X
R109	(K)	K		X
R155	G,I,K,L,M,Q,S,T	G,I,K,L,M,Q,S,T	X	X
A156	I,S,T,V	I,S,T,V	X	X
D168	A,E,G,N,V	A,E,G,H,N,V	X	X
V170/I170	A,L,T	A	(X)*	X
E176		G		X

*Variant only found in vitro.

TEL; telaprevir, BOC; boceprevir.

Phylogenetic analysis of the NS3 sequences was performed with the Neighbour-Joining method, using Kimura 2-parameter as the nucleotide substitution model (PHYLIP, v3.6). The tree was visualized with TreeView (**Fig. 1**). Molecular evolution analysis of the NS3 quasispecies in each patient was performed with MEGA software (version 5) [19].

### Statistical analysis

Data is presented as absolute numbers. Categorical data was analyzed with Pearson's Chi Square test and continuous data with Fisher's exact test. A significant confidence interval at 95% was used. Rstudio software was used for all statistical analysis (www.R-project.org).

### Ethical considerations

All patients signed written informed consent before inclusion in the study and storage of their samples at the DANHEP biobank. The Danish Research Ethics Committee (H-1-2011-139) and The Danish Data Protection Agency (J nr 2011-41-6542) approved the study.

## Results

### Patient Characteristics

**Figure 2:**
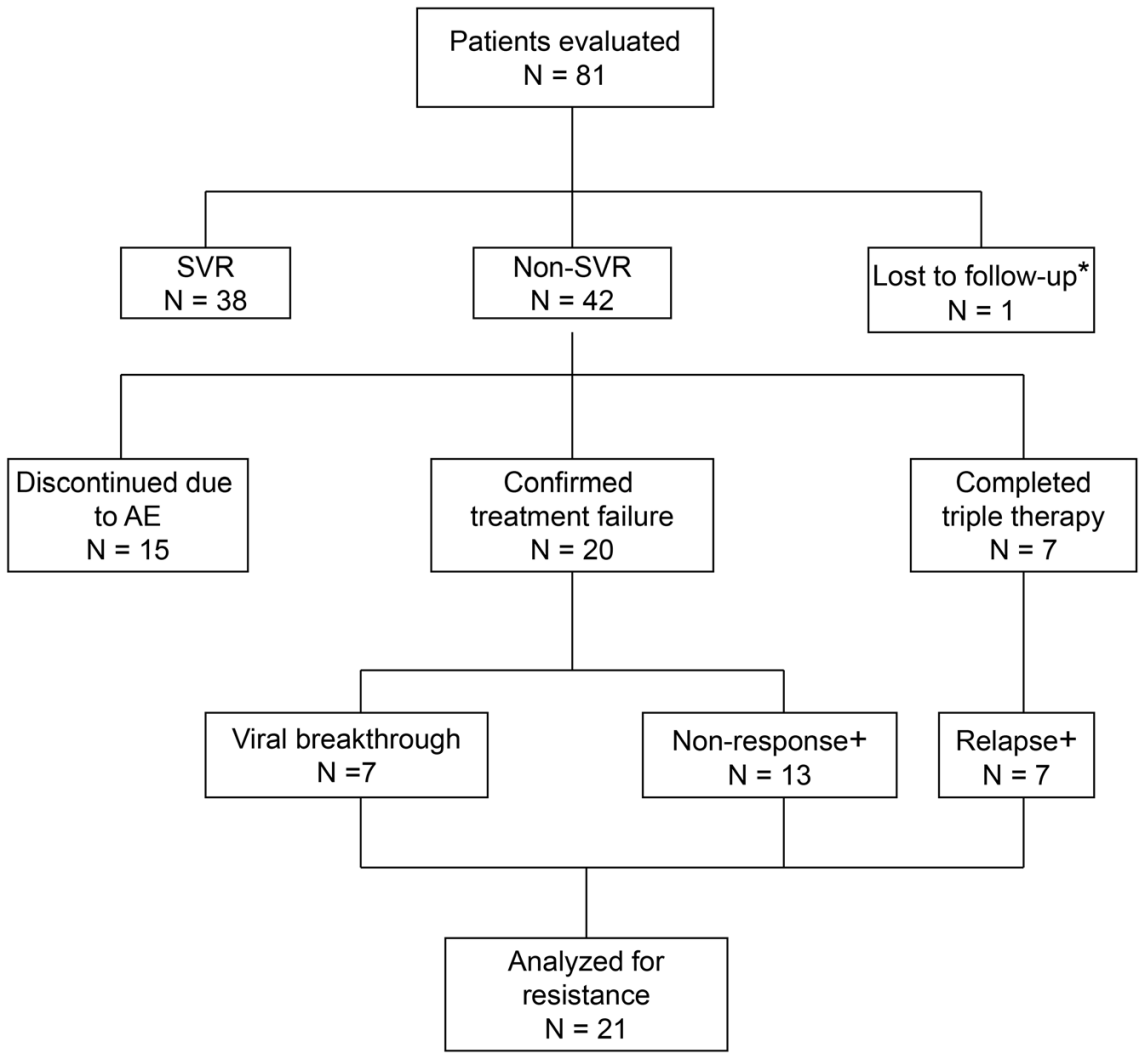
Flowchart. ^*^The patient was lost to follow-up at treatment week 16. ^+^Six patients (3 with non-response, 2 with relapse and 1 with HCV GT 1l) were not analyzed for resistance mutations.

In Denmark, 80 patients with chronic HCV genotype 1 infection initiated and completed triple therapy during the study period. Demographic characteristics and treatment outcome are shown in **Table 1** and **Fig. 2**. Fifty-three (66%) and 26 (33%) patients had HCV GT 1a and 1b, respectively; a single patient had genotype 1l.

BOC was used in 42 (53%) and TEL in 38 (47%) of the patients. RBV dose reduction was seen in 32 (40%) patients. All patients with SVR received minimum 50% of the standard amount of RBV but in two patients RBV was discontinued due to anaemia. In the non-SVR group, three patients (7%) received <50% of RBV assigned by response-guided therapy and three patients were taken off RBV during treatment.

### Virological Response

**Figure 3:**
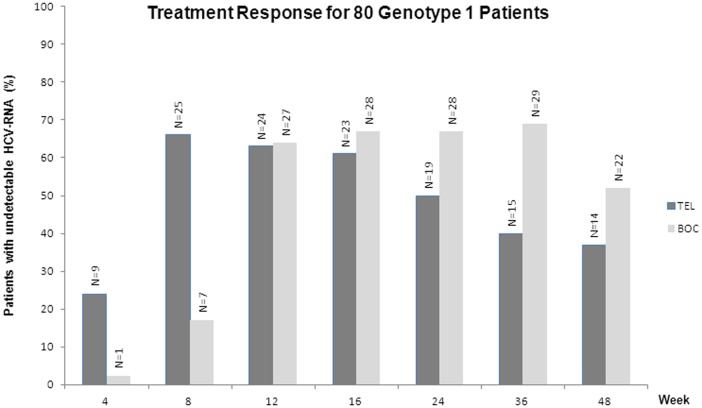
Treatment response for 38 TEL-treated and 42 BOC-treated patients.

Thirty-eight (47%) patients achieved SVR (**Table 1** **and** **Fig. 2**). **Figure 3** shows treatment responses for TEL and BOC, expressed as the percentage of patients with undetectable HCV-RNA during treatment weeks 4 to 48.

Only 1 (2.4%) BOC treated patient had undetectable HCV-RNA at week 4, which could be explained by the 4-week lead-in phase with PEGINF/RBV. Peak TEL response (25 patients (66%) with undetectable HCV-RNA) was reached at week 8, but thereafter the number of patients with undetectable HCV-RNA decreased. For BOC, the number of patients with undetectable HCV-RNA increased to 29 (69%) at week 36. At week 48 it dropped to 52% due to either relapse or viral breakthrough.

Among patients with SVR, 4 discontinued treatment due to adverse events. One was treated with BOC (week 7 (n = 1)) and 3 with TEL (weeks 7 (n = 1), 14 (n = 1) and 22 (n = 1)). Fifteen of the 42 patients with non-SVR, including 3 BOC-treated (weeks 4 (n = 1), 8 (n = 2)) and 12 TEL-treated (weeks 1 (n = 1), 2 (n = 2), 5 (n = 1), 6 (n = 4), 8 (n = 2), 10 (n = 1) and 13 (n = 1)), discontinued treatment due to adverse events (anaemia, thrombocytopenia, skin rash or poor general condition).

### Viral Sequence Analysis Reveals NS3 protease changes post-treatment

The NS3 protease sequence of HCV recovered from 10, 4 and 7 non-responder, relapse and viral breakthrough patients, respectively, were analyzed (**Fig. 1**). Thirteen, 4 and 4 patients had cirrhosis, moderate fibrosis, and mild fibrosis, respectively, prior to treatment initiation. Seven (33%) were treatment-naïve and 14 (67%) were non-responders/relapsers to previous PEGINF/RBV treatment. Eleven (52%) patients received TEL; 10 (48%) BOC.

To determine whether changes in the protease domain of NS3 had occurred after triple therapy we compared the viral direct sequence in the pre- and post-treatment samples (**Table 5**). Additionally, in a selected group of 14 patients, we performed clonal analysis (10 clones per sample) in both pre- and post-treatment samples (**Table 6**).

**Table 5:** Amino acid changes present after triple therapy with TEL and BOC in 21 patients without SVR.

Patient ID	GT	TEL/BOC treatment	Amino acid changes* (Protease)	Triple therapy completed (week)	Post-treatment analysed (week)	HCV-RNA titre Pre-treatment (IU/ml)	HCV-RNA titre Post-treatment (IU/ml)	Virological Response
1	1a	TEL	**R155K**, *A40T*	40	40	5320000	24100	BT
2	1a	TEL	**V36M, I170T**	24	24	82700	2390	BT
3	1a	TEL	**R155K**	24	36	5300000	570000	RL
4	1a	TEL	**V36M, R155K**, *V150A, V29A*	8	8	2600000	4600	NR
5	1a	TEL	**V36M, R155K**	16	24	23000000	2700000	BT
6	1a	TEL	**V36M, R155K,** *V147A,*	12	12	10000000	2300	NR
7	1a	TEL	**T54S, R155K**	8	64	2300000	270000	NR
8	1a	BOC	**V36M**, *S20N, P88L*	12	12	900000	500000	NR
9	1a	BOC	**R155K,** *R129P*	24	40	2500000	3400000	NR
10	1a	BOC	S122G	24	48	12000000	2300000	NR
11	1a	BOC	**V36M**, *I170V* ++, *P67S*	12	12	48000000	4800	NR
12	1a	BOC	**Q80L** +, *I83V*	24	48	2200000	14000000	RL
13	1a	BOC	*V153L*	28	29	280000	430000	RL
14	1a	BOC	None	16	64	1400000	3800000	NR
15	1b	TEL	**A156T**	5	5	2200000	670000	NR
16	1b	TEL	**V36A**, *L13V*	16	16	139000	249000	BT
17	1b	TEL	**V36A**, *I18V, V48I*	16	16	6200000	100000	BT
18	1b	TEL	None	24	24	5790000	174000	BT
19	1b	BOC	**(V55A** ** **)**	16	40	630000	330000	NR
20	1b	BOC	*A150V, P89S, S101N*	48	60	20000000	1900000	RL
21	1b	BOC	None	48	96	6500000	440000	BT

*Letter on the left represents the wild type amino acid (aa), on the right, the aa substitution. Amino acid changes known to confer resistance to PI's are reported in bold and other observed sequence variants are shown in italics.

**Clonal analysis revealed 1 clone with the resistance mutation V55A.

+ Only found as a minor quasispecies pre-treatment.

++ I170V has been described as a polymorphism not associated with resistance in GT3.

GT; genotype, BT; Breakthrough. NR; Non-response. RL; Relapse.

**Table 6:** Clonal analysis of the NS3 protease in 14 non-SVR patients.

AA in NS3	Patient ID-change													
	1	2	3	4	5	8	9	10	11	15	16	17	18	19
13											L-V (100)			
20						S-N (100)								
21													L-P (20)	
33		V-I (50)												
**36**		V-M (100)		V-M (100)	V-M (100)	V-M (100)			V-M (100)		V-A (50)	V-A (100)		
40	A-T (80)													
**55**														V-A (10)
67									P-S (30)					
75								Y-N (20)						
88						P-L (100)								
122									S-G (20)					
132		P-V (50)												
**155**	R-K (100)		R-K (100)	R-K (100)	R-K (100)		R-K (90)							
**156**										A-T (100)				
**170**		I-T (50)							I-V (70)					
174			N-S (20)											
184								F-I (20)						

Clonal analysis of the protease domain of NS3 in selected patients. Ten clones from the pre- and the post-treatment samples were analyzed and the changes that appeared in the post-treatment sample are depicted in the table. The percentage of clones containing the specified change is indicated in parenthesis. Only changes present in more than 1 clone were taken into account, except for patient 19, where the resistance variant V55A is represented even though there was only 1 clone out of 10 that contained the change. Amino acid positions previously reported as involved in resistance to PI's are highlighted in bold.

### Viral resistance in genotype 1a

We identified NS3 protease changes in 13 of 14 patients with GT1a; changes were not detected in one patient (Pt. 14). In all patients, except one (Pt.12), showing amino acid changes in the post treatment sequence, those changes were not present in the pre-treatment sequence. Among the observed aa changes, positions V36, T54, Q80, R155 and I170 were previously involved in resistance to PI's (**Table 4**) [5]–[7], [20]. The combination of V36M and R155K, known to confer high level of PI resistance, was present in patients 4, 5 and 6 post-treatment. Clonal analysis of post-treatment samples from patients 4 and 5, where all clones contained the combination V36M/R155K, further supported these results. The single resistant variant R155K was present in patients 1, 3 and 9 post-treatment; clonal analysis confirmed R155K in all three patients. V36M as a single resistance variant was present post-treatment in patients 8 and 11. Patients 10 and 13 presented only changes in residues S122 or V153 post-treatment, respectively, which have not been previously reported as resistant variants against PI's (**Table 4**). In patient 10, the clonal analysis did not show the change S122G but changes Y75N (20% of clones) and F184I (20% of clones). Patient 12 showed Q80Q/L, previously reported as a resistant mutation, in the direct sequence of both the pre- and post-treatment sample. In patient 2, two resistance variants, V36M and I170T were detected in the direct sequencing of the post-treatment sample; V36M appeared in all clones post-treatment, while I170T was present in only 50% of the clones. The post-treatment sample of patient 7 showed the resistance variants T54S and R155K.

### Viral resistance in genotype 1b

The direct NS3 sequences of HCV recovered from the seven non-SVR patients with GT1b, revealed 2 patients (patients 18 and 21) with no changes in the protease domain sequence pre- or post-treatment (**Table 5**), but the remaining patients showed changes that were not present in the pre-treatment sample. Patient 19 had a unique clone in the post-treatment sample containing the resistant variant V55A. Patients 15, 16, and 17 each had a single previously reported resistant mutation (A156T, V36A, and V36A, respectively) post-treatment; the clonal analysis agreed with the direct sequencing in all three patients. Patient 20 presented three changes post-treatment not previously involved in resistance to PI's.

### Quasispecies analysis

For each of the 14 non-SVR patients analyzed, nucleotide clonal sequences were divided into pre- and post-treatment groups and diverse population parameters were calculated. Intra and inter-population genetic distances were estimated for each patient (**Table 7**). For 5 of 9 GT1a patients and 3 of 5 GT1b patients, genetic distances between sequences were higher in the pre-treatment population than in the post-treatment population, suggesting a decrease in diversity of the quasispecies after treatment. We observed no relationship between HCV GT or type of PI to the increase or decrease in genetic diversity of HCV.

**Table 7:** Genetic diversity and dN/dS ratio for 14 non-SVR patients selected for clonal analysis of the NS3 protease gene.

				Genetic diversity		dN/dS
Patient ID	GT	PI	Pre	Post	Pre/Post	Pre/Post
1	1a	TEL	0,009	0,017	0,031	0,094
2	1a	TEL	0,005	0,016	0,048	0,048
3	1a	TEL	0,016	0,005	0,02	0,071
4	1a	TEL	0,024	0,003	0,047	0,063
5	1a	TEL	0,022	0,005	0,018	0,303
8	1a	BOC	0,012	0	0,014	0,417
9	1a	BOC	0,006	0,007	0,014	0,098
10	1a	BOC	0,01	0,002	0,014	0,054
11	1a	BOC	0,001	0,011	0,015	0,194
15	1b	TEL	0,007	0,005	0,01	0,174
16	1b	TEL	0,002	0,012	0,015	0,176
17	1b	TEL	0,012	0	0,016	0,290
18	1b	TEL	0,001	0,003	0,017	0,035
19	1b	BOC	0,045	0,027	0,05	0,024

GT; genotype.

PI; protease inhibitor.

SVR; sustained viral response.

dN/dS <1; dN; Non-synonymous mutation substitution rate, dS; synonymous mutation substitution rate <1 suggest purifying selection.

The dN/dS ratio was calculated for the 14 patients to define the direction and magnitude of natural selection acting on the protein coding genes.

Overall, the number of synonymous substitutions per synonymous site (dS) exceeded the number of non-synonymous substitutions per non-synonymous site (dN) and as previously reported [21], [22], a dN/dS ratio <1 was observed in all patients suggesting that purifying selection was acting in the NS3 protease domain (**Table 7**).

**Figure 4:**
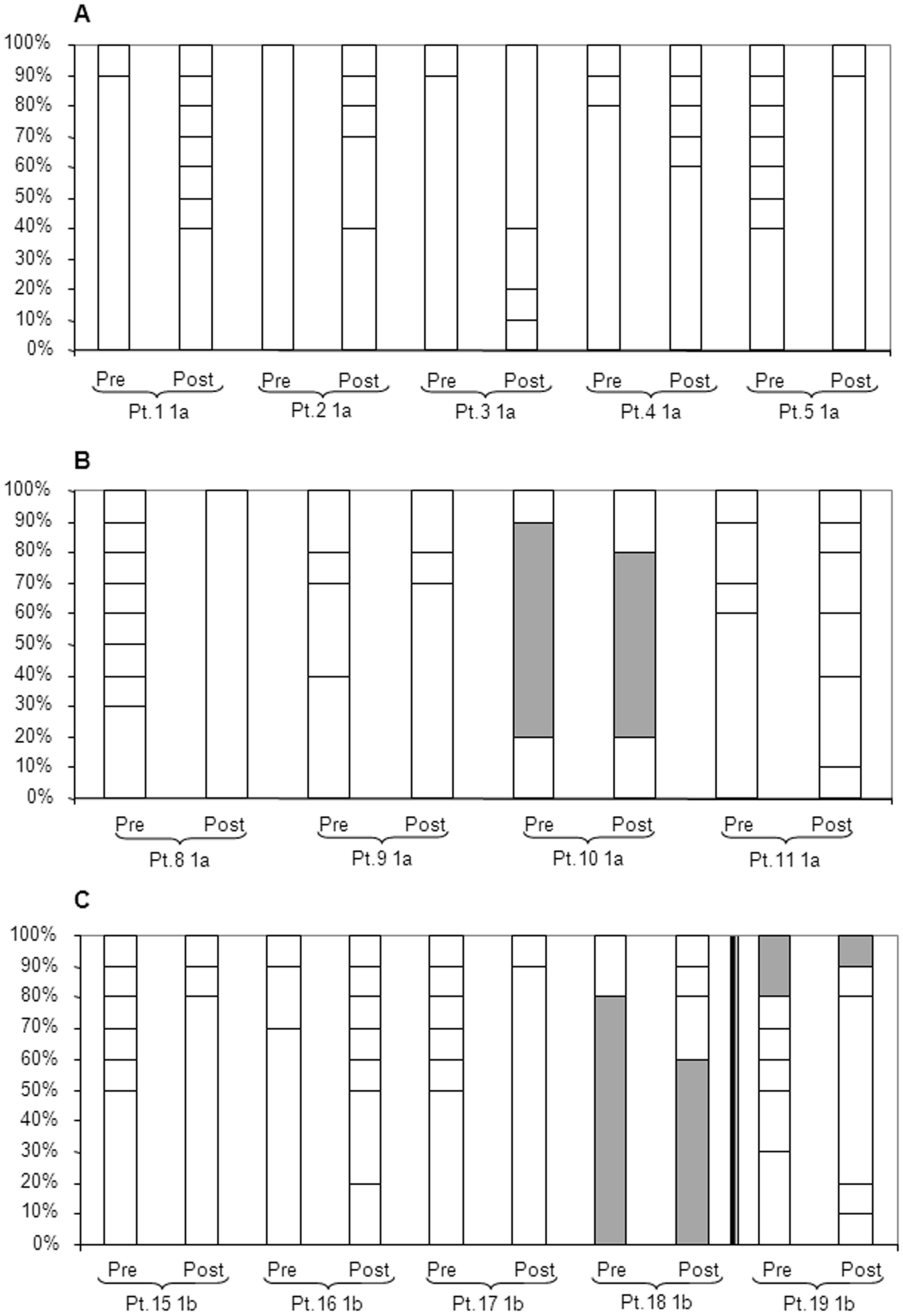
Number of variants comprising the NS3 protease domain quasispecies (amino acid) in pre- and post-treatment samples in 14 non-SVR patients. Vertical bars: number of viral variants/sample. Y-axis: percentage of each sequence in respect to the total population (10 clones). Panel A: TEL-treated GT1a patients (n = 5). B: BOC-treated GT1a patients (n = 4), C: TEL- (n = 4) and BOC-treated (n = 1; Pt. 19) GT1b patients. Shadings indicate identical clonal sequence in pre- and post-sample.

We also determined the number of variants at the aa level in the quasispecies pre- and post-treatment. In GT1a patients, the number of different variants increased in the post-treatment quasispecies in four of the five TEL-treated patients. In GT1a BOC-treated patients, the number of clones with aa differences only increased post-treatment in patient 11, remained unchanged in patient 10 and decreased in patients 8 and 9. Regarding GT1b infected patients, the number of different clonal variants decreased after treatment in three patients and increased in the remaining two (**Fig. 4**).

## Discussion

The present study is to our knowledge the first to report SVR rates at week 24 after triple therapy as well as detection of resistance mutations in patients with chronic hepatitis C GT1 in a nationwide, routine clinical setting. The SVR rate was only 47% and well-described resistance variants were detected in the majority of non-SVR patients. An explanation for the much lower SVR rate than the SVR rate reported in clinical trials [1], [23] could be that study populations in clinical trials are usually highly selected. The SVR rate in our study was similar to SVR rates reported in patients treated with PEGINF/RBV [24].

From the French Early Access Programme including 497 PEGINF/RBV treatment-experienced patients with HCV GT1 infection and compensated cirrhosis [25], response rates at post-treatment week 16 have been reported to be 67% for TEL-treated and 58% for BOC-treated patients, respectively. However, as SVR rates have not yet been reported and the patient characteristics of the French cohort differ significantly from ours, direct comparison is not possible. A recent study by Macartney et al. [26] including 58 treatment naïve and experienced patients found that 37 (63.8%) had undetectable HCV- RNA 12 weeks after EOT. However, SVR rates at week 24 have not been published and may be lower due to relapse during the follow-up period.

The poor response to triple therapy in our study could partly be explained by a significantly larger number of patients with cirrhosis in the non-SVR – versus the SVR group (23 (70%) versus 10 (30%)), as well as fewer patients with the favourable IL28-B genotype CC (4 (29%) versus 10 (71%)). In the study by Maasoumy et al., IL-28B genotype CC was present in 18% of patients which is similar to our results with 14 (21%) of 67 patients and was also associated with a better treatment outcome.

We observed that adverse events leading to early treatment discontinuation occurred in 20 (25%) patients, similar to observations from other non-clinical trials [25], [27]. Poordad et al. [28] observed that RBV dose reduction throughout the course of triple therapy did not affect SVR rates, as long as the patients received at least 50% of total amount RBV assigned by response-guided RBV therapy. We observed no difference between SVR (42%) and non-SVR (38%) patients in relation to RBV reduction.

We detected well-described PI resistance variants in 15 (71%) of 21 patients analyzed. A similar rate (50–75%) has been shown in patients in phase III trials at time of triple therapy failure [18] and a rate of 81% was seen in a routine clinical setting in the study by Macartney et al. [26]. In consistency with other studies [8], [18], [20], the most frequently detected variants in patients with HCV GT1a infection were R155K and V36M, whereas for GT1b infection, V36A was mainly observed.

Interestingly we did not detect Q80K, the signature mutation for the recently licensed PI simeprevir, neither at baseline nor at treatment failure. This indicates that Q80K may not be as prevalent in Denmark as in the US where 20% of the population harbours this mutation at baseline [18]. This may also suggest that patients who failed 1^st^. generation PI treatment may not be resistant to simeprevir.

Twelve patients showed amino acid changes in the post treatment sample that had not been previously associated with resistance to PIs. To our knowledge, from these changes, only amino acid P88 (seen in patient 8), have been previously described in the literature, and was associated with viral fitness compensation. The function of most of the additional changes reported in this manuscript is yet to be defined in future studies.

While one patient (Pt. 15) had A156T, which is known to confer the highest level of resistance to TEL and BOC [8], six of the non-SVR patients in this study had no known resistance variants detected pre- or post-treatment. We cannot discard that resistant mutations were indeed present in a small portion of the viral population, and therefore could not be detected by direct sequencing or clonal analysis of 10 clones in the indicated cases. Other methodologies such as deep sequencing could help clarifying this matter. On the other hand in four of these six patients, post-treatment analysed samples were obtained more than 10 weeks after EOT, which could imply, that in most of the circulating variants carrying resistance mutations these mutations would have reverted to the original amino acid, due to the decrease in viral fitness they produce. In this regard, studies have shown that the frequency of PI resistance variants decline over time, following drug withdrawal, but in some cases, resistance variants can be found at low-level in post-treatment samples, up to five years after treatment completion, using high resolution deep sequencing analysis [2], [3], [8].

PI's in phase II and III trials show extensive cross-resistance with TEL and BOC [18], [20]. Patients with detectable resistance variants included in this study should be tested short-term and long-term for the persistence of resistance variants as it is currently unknown, if the found variants can re-emerge during re-treatment with 2^nd^ generation PI's.

Regarding the viral quasispecies analysis in non-SVR patients, no clear pattern of genetic evolution was observed. The lack of correlation with clinical data could be a result of the complexities of the forces driving evolution of NS3. The NS3 protease domain contains immunological relevant epitopes and therefore is subjected to immune selection, as seen in natural evolution of NS3 in chronically infected patients [29]. In patients treated with PI, a second evolutionary force takes action, and at last both selective pressures may act together, since the immune epitopes may overlap with PI resistant mutations [22], creating a complex interplay.

After treatment, the viral population (amino acid level) changed significantly. In the post-treatment viral population, most patients presented sequences that were different from the sequences found in the pre-treatment population. Only three patients had post- treatment sequences that were already found in the pre-treatment sample. Interestingly, these patients had none or very low frequency of resistant mutations after treatment thus indicating a trend towards preservation of pre-treatment sequences through the post-treatment in the absence (or very low frequency) of PI resistant mutations. However, due to the small number of patients these findings remain to be confirmed in larger studies.

As previously observed in the analysis of NS3 [21], [22], the dN/dS was less than 1, suggesting purifying selection in the protease sequence during triple therapy. This result was unexpected, since most of the patients had a dramatic change in the quasispecies, where a minor variant harbouring a resistance mutation increased its frequency in the quasispecies and became a dominant sequence after treatment, thus suggesting strong positive selection. Domingo et al. [30] have reported this phenomenon, where a few amino acid changes driving the evolution of a region are accompanied by an excess of synonymous changes, greatly affecting the results of the dN/dS ratio.

The strengths of this study is its nationwide conduction and the continuous recording of patient data and blood samples into DANHEP, making it possible to obtain data on demographic characteristics and treatment response on all patients receiving triple therapy outside clinical trials in Denmark. Our lost to follow-up was limited to one patient who discontinued treatment after 16 weeks.

There are some limitations to the present study. Denmark is a relative small country with a low prevalence of chronic HCV GT1 infected patients, affecting the number of patients possible to treat with triple therapy. Due to the DANHEP database and the close collaboration between the treating departments, the 81 patients included in this study represent the total number of patients who started triple therapy in Denmark during the study period and before treatment with 1^st^ generation PI's stopped due to the possibility of treating with 2^nd^ generation Direct Acting Antivirals (DAAs). The compliance to drug intake was reflected in the blood samples, with decreased levels of neutrophilocytes, platelets and red blood cells, in patients undergoing treatment and was rated to be very good by the treating physicians. Only 4 (5%) patients had one missed blood sample during the treatment course which reflects the very good show up and adherence to treatment at the different departments. We used clonal analysis in 14 patients, which is a recommended procedure for detection of complex mutation patterns, but the sensitivity of the procedure is highly dependent on the number of sequenced clones. Clonal sequencing can detect variants at very low frequencies, requiring a sequence rate >80 clones for a 4% detection limit at a 95% confidence level [20]. Ten clones were analyzed in this study, which is a limiting factor in detecting variants found pre-treatment at a very low level. Studies using deep sequencing indicate that pre-existing protease resistant variants are detectable at the 1% level in approximately 7–8% of patients [18]. This method could perhaps have increased the detection rate of resistance variants at baseline and could have clarified if patients with non-SVR already pre-treatment had the resistance variants found later. However, we found a very good correlation between the direct sequencing and the clonal analysis. In only two patients did clonal analysis reveal a resistance mutation not detected by direct sequencing (patients 2 and 19), and in five patients, aa changes, unrelated to resistance, arose after clonal analysis.

In conclusion, this study is the first to report, on a nationwide basis, that SVR rates after triple therapy outside clinical trials was lower than that in clinical trials and comparable to the SVR rate obtained by PEGINF/RBV therapy. The overall success rate of these new treatment regimens towards chronic HCV GT1 infection will require more data from routine clinical settings. Furthermore, we detected no major pre-treatment sequence variants associated with resistance to PI's, but during treatment, resistance variants occurred in the majority of patients with non-SVR. These data are important in relation to future treatment with 2^nd^ generation PI's.
